# Health care worker carriage of drug-resistant bacteria and infection control practices at a tertiary care hospital in Uganda: a cross-sectional survey

**DOI:** 10.11604/pamj.2023.45.68.36315

**Published:** 2023-05-30

**Authors:** Daniel Bulwadda, Francis Kakooza, John Paul Waswa, Henry Bosa Kyobe, Moses Sembatya, Reuben Kiggundu

**Affiliations:** 1 Department of Medical Microbiology, School of Medicine, Makerere University, Kampala, Uganda,; 2 Infectious Diseases Institute, Makerere University, Kampala, Uganda,; 3 Department of Immunology and Molecular Biology, Makerere University College of Health Sciences, Kampala, Uganda,; 4 USAID Medicines, Technologies, and Pharmaceutical Services (MTaPS) Program, Management Sciences for Health, Kampala, Uganda,; 5 Management Sciences for Health, Kampala, Uganda

**Keywords:** Antimicrobial resistance, antibiotics, drug-resistant, pathogenic bacteria carriage, infection prevention and control, health care worker

## Abstract

**Introduction:**

bacterial carriage by health care workers (HCWs) is a major risk factor for transmission of healthcare-associated infections (HAIs). Often, these pathogens are multiple drug resistant (MDR) and are transmitted from hospital environments. We aimed to study the carriage of pathogenic bacteria among HCWs at a tertiary care hospital in Uganda.

**Methods:**

a cross-sectional study was done at Naguru Regional Referral Hospital from June 2017 to August 2017. Five finger imprints of both hands-on blood and MacConkey agar were done. We assessed pathogenic bacterial carriage by HCWs and characterized drug sensitivity and relatedness of these isolates. Genotyping of extended-spectrum beta-lactamase (ESBL) and Methicillin-resistant Staphylococcus aureus (MRSA) positive isolates was done to determine intra-hospital transmission. A survey of the hospital’s IPC de program was done.

**Results:**

one hundred and eight (108) HCWs were enrolled. Carriage of pathogenic bacteria was highest in surgical and emergency wards at 36% and 35.6% respectively, p-value of 0.00. The proportion of microbial carriage was highest among nurses 16 (34.8%) followed by medical officers 11 (23.9%). Among the isolated pathogenic bacteria, 25 (36.2%) were Gram-positive and 44 (63.8%) were Gram-negative. Fifty percent of Staphylococcus aureus were methicillin-resistant, and one isolate was vancomycin-resistant. Fifty-four percent (54.6%) of HCWs had never been trained on moments of hand hygiene, only 44.4% recognized the presence of an IPC program in the hospital and 49% were not aware of problems associated with poor IPC practices.

**Conclusion:**

this study demonstrated that hands of HCWs at Naguru Regional Referral Hospital were colonized with pathogenic bacteria with varying prevalence, some with multidrug-resistant strains including MRSA and ESBL.

## Introduction

Healthcare-associated infections (HAIs) are those acquired during the process of health care and represent a major cause of morbidity and mortality. The World Health Organization (WHO) estimates the prevalence of HAIs at 10-15%, with a higher prevalence in resource-limited settings [1]. The prevalence is even higher among vulnerable patients including those admitted to intensive care units (ICUs), in which the prevalence of HAIs may be as high as 50% in resource-limited settings [2]. Disappointingly, poor diagnostic and surveillance systems leave many cases of HAIs in sub-Saharan Africa unreported [1].

Often, HAIs are caused by multiple drug-resistant organisms, and this greatly increases the risk of adverse treatment outcomes including therapeutic challenges, prolonged hospitalizations, morbidity, mortality, and higher costs of health care. Routine surveillance of HAIs is the cornerstone to understanding and addressing this emerging public health threat. Variations in causative organisms and sensitivity patterns for HAIs can occur in the same health facility [3,4]. A survey of causes of HAIs among patients admitted to the surgical ward at Mulago Hospital showed that 23.7% were due to *Escherichia coli (E. coli)*, 21.1% *Staphylococcus aureus*, and 17.1% *Acinetobacter species* (22%) as the causative pathogens [5]. A survey among patients admitted to the ICU at the same hospital showed that *Klebsiella pneumonia* (30%), *Acinetobacter species* (22%,) and *Staphylococcus aureus* (14%) were the most frequently isolated bacteria [6].

Acquisition of an infectious agent is aided by 3 factors including; 1) the source of the organism, 2) the presence of a susceptible host, and 3) the mode of transmission of the infectious agent [7]. Contaminated hospital environments are a known source of pathogenic bacteria and the spread of HAIs [8]. In many cases, HCWs act as the vector for the spread of infections to patients from the hospital environment or from patient to patient. This process is aided by poor hand hygiene practices [8]. Breaking this chain of bacterial transmission is the focus of HAI control efforts [9].

Despite hand hygiene being an effective and cheap method for preventing the spread of HAIs, compliance remains, low, especially in resource-limited settings [10]. Factors contributing to low hand hygiene compliance include overcrowding, understaffing, lack of training, and lack of IPC policies and guidelines in health facilities [11]. Putting all these variables, this study aimed at assessing HCW carriage of drug-resistant bacteria and infection prevention and control (IPC) practices at a tertiary care hospital in Uganda. The objective of the study was to determine the extent of pathogenic and multi-drug resistant bacteria hand carriage among HCWs at Naguru Hospital.

## Methods

**Study participants:** one hundred and eight (108) HCWs at Naguru Regional Referral Hospital provided written informed consent to participate in the study from June 2016 to August 2016. Health care workers were classified as cleaners, nurses, clinical officers, medical doctors, and “others” (security personnel, pharmacists, and administrators). Naguru Hospital is a 100-bed capacity public hospital that provides both primary and tertiary care mainly to people living around Kampala, Uganda. The sample size was calculated using the Kish Leslie 1965 method. We assumed a 50% carriage of pathogenic bacteria by health workers when calculating the sample size. We included health workers who: were actively involved in direct contact with patients, had been working at inpatient units for the last 48 hours and were usually in contact with the hospital environment (i.e., were on duty for more than 4 days a week). We excluded those that were on antibiotic treatment, those with visible wounds on their hands and those found with visibly dirty hands.

**Sample size calculation:** sample size estimation was done using Kish Leslie 1965:


$$N=\frac{Z^{2}p\left(1-p\right)}{d^{2}}$$

Where; Z= Z score for 95% confidence interval= 1.96. P= 50% prevalence since the prevalence of HCWs' hand pathogenic carriage was not known. N= number of HCWs who participated in the study, and d= tolerable error = 5%. Hence:


$$n=\frac{{\left(1.96\right)}^{2}*0.5\left(1-0.5\right)}{0.05^{2}}=384$$

I adjusted the sample size due to cost-time implications and the HCWs population at Naguru Hospital (150 HCWs) accordingly to this Kish Leslie formula:


$$S=\frac{N}{1+N/population\text{ size}}$$

Where S= adjusted sample size and N= number of HCWs who participated in the study. Therefore, we used a sample size of 108 study participants:


$$S=\frac{384}{\left(1-384/150\right)}=107.8\approx 108\;study\;participants$$

**Demographic data:** data about participant´s demographics, knowledge, attitudes and practices for hand hygiene and the presence of infrastructure for the IPC program were obtained using a structured questionnaire.

**Sample collection, transportation, and laboratory procedures:** sample collection, transportation and processing were guided by standard practice [12]. The sample collection procedure was explained and demonstrated by the research officer to each participant on how to inoculate the media plates with his or her fingertips. The research officer used aseptic techniques during the collection of samples to avoid contamination of culture media. Inoculation was done by taking imprints of five fingertips of both hands on both blood and MacConkey agar respectively. During the process of sample collection, fingertips were gently pressed for five seconds on the culture media. The inoculated samples were transported to the medical microbiology laboratory within 2 hours following standard microbiology sample transportation procedures.

**Bacteriology testing:** inoculated blood and MacConkey agar were then incubated at 35-37°C for 18-24 hours in carbon dioxide and ambient air incubators, respectively. Following incubation, purity plating was done according to colony morphology, haemolysis on blood agar and lactose fermentation on MacConkey agar. Then overnight incubation of purity plated media was done at 35-37°C. Identifications were done according to conventional physiological and biochemical methods such as Gram stain, catalase reaction, Mannitol salt, coagulase test, DNase test, haemolytic activity on blood agar, bacitracin, Optochin and SXT antimicrobial identification discs and bile Esculin test for Gram-positive bacteria as described in the standard operating procedures of clinical microbiology bacteriology laboratory. Gram-negative bacteria were identified based on colony morphology and lactose fermentation on MacConkey agar, then followed by biochemical reactions namely, oxidase, Triple Sugar Iron (TSI), Sulphur Indole and Motility (SIM), citrate and urease tests.

**Drug susceptibility testing:** drug susceptibility tests were done by use of the standard disc diffusion technique as recommended by the Clinical and Laboratory Standard Institute (CLSI) [13]. For Gram-positive bacteria, discs (Biolab®, HUNGARY) used were ampicillin (10μg), cefoxitin (30μg), trimethoprim-sulphamethoxazole (1.25/23.75μg), tetracycline (30μg), ciprofloxacin (5μg), chloramphenicol (30μg), gentamicin (10μg), erythromycin (15μg), clindamycin (2μg), and vancomycin (30μg). Phenotypic screening for MRSA was done on all identified *S. aureus*, using cefoxitin (30μg) disc diffusion zones less than or equal to 19 mm were reported as oxacillin resistant. Those for which cefoxitin zones were greater than or equal to 20 mm were reported as oxacillin susceptible. For gram negative bacteria discs (Biolab®, HUNGARY) used; ampicillin (10μg), piperacillin (100μg), piperacillin-tazobactam (100/10μg), amoxicillin-clavulanic acid (20/10μg), trimethoprim-sulphamethoxazole (1.25/23.75μg), tetracycline (30μg), ciprofloxacin (5μg), chloramphenicol (30μg), gentamicin (10μg), amikacin (30μg), ceftriaxone (30μg), ceftazidime (30μg), cefepime (30μg) and imipenem (10μg). Screening test for resistance types was carried out for ESBL-producing organisms using the double disc synergy method; carbapenemase-producing organisms using the modified Hodge´s test, a combination of meropenem with EDTA for screening metallo-beta lactamases type carbapenemase (MBL) and boronic acid for *Klebsiella pneumonia* carbapenemase (KPC).

**Detecting transmission of bacteria among health care workers**

**Deoxyribonucleic acid (DNA) extraction:** deoxyribonucleic acid for amplification was extracted from whole cells by the boiling lysis method as explained briefly below. A full loop of pure colonies from fresh pure cultures was suspended in 1 ml of sterile distilled water. The cells were lysed by heating at 95°C for 10 minutes. The cells were later vortexed for 5 seconds. Centrifugation was later done at 13,000 rpm for 5 minutes and kept at -20°C to harvest the supernatant containing the DNA. The supernatant was subsequently used to do PCR and used as template DNA. The integrity of extracted DNA was evaluated by electrophoresis on 1% agarose gel. Also, the purity of DNA was determined by the ratio A260/A280 in a biophotometer.

**Enterobacterial repetitive intergenic consensus polymerase chain reaction (ERIC-PCR) amplification of extended-spectrum beta-lactamase *E. coli*:** enterobacterial repetitive intergenic consensus polymerase chain reaction was carried out following the principles of the methods previously based on a report by Versalovic *et al*. [14]. Briefly, a 20μL PCR reaction was prepared using the Hotstar PCR reaction kit (QIAGEN, Germany) following the manufacturer´s recommendations. Each tube contained a mixture of 9.0μL of PCR water, 2.0μL of 10xPCR buffer, 4.0μL of Q-solution, 1.0μL of 10mM MgCl2, 0.8μL of 10mM dNTPs, 0.5μL of each of the ERIC Reverse (5'-ATGTAAGCTCCTGGG GATTCAC-3') and Forward (5'-AAGTAA GTGACTGGGGTGAGCG-3') Primers, and 0.1μL of 5U/μL of Taq DNA polymerase. Finally, 2.0μL of *E. coli* DNA was added to each tube to obtain a 20μL volume. The PCR tubes were loaded in a Thermocycler (Bio-Rad Laboratories Inc., Singapore) to run an amplification profile.

**Agarose gel analysis:** polymerase chain reaction products were analyzed using 3% w/v Agarose gel (Fisher Scientific, USA), in 1X Tris Acetate EDTA (TAE) buffer, stained with 5mg/ml of Ethidium Bromide (Sigma-Aldrich, USA). The gels were run at 100V for a minimum of 5 hours and visualized using a UVP Gel documentation system (Benchtop Trans-Illuminator System -BioDoc-it, CA, USA).

**Methicillin-resistant *Staphylococcus aureus* spa genotyping and deoxyribonucleic acid sequencing:** to determine the genetic lineages of *S. aureus*, spa genotyping was performed, and the X-region of the spa gene was amplified by PCR primers Spa Forward (5'-AGA CGATCCTTCGGTGAG-3') and spa reverse (5'-GCTTTTGCAATGTCATTTACTG-3') Primers. The amplified PCR product (~15.0μL) was purified using the *QIAmp DNA purification mini kit* (QIAGEN, Germany) following the manufacturer´s guidelines. Purified amplicons were sequenced using an automated sequencer; the Sanger platform (ACGT, Wheeling, USA) with standard commercial kits, following the manufacturer´s recommendations. Then determining the spaTyper, the sequences were submitted to the spaTyper database [15] and the data was crosschecked with the spa database (Ridom GmbH, Wurzburg, Germany) [16].

**Quality of laboratory assays:** to ensure that the results were valid and reliable, control strains were used alongside the isolates from the specimen. These included *E. coli* ATCC 25922, *E. coli* ATCC 35218, *P. aeruginosa* ATCC 27853, *S. aureus* ATCC 25923, *E. faecalis* ATCC 51299, *E. faecalis* ATCC 29212, *K. pneumonia* ATCC 700603 *K. pneumonia* ATCC BAA-1705 and 1706. These strains were used to quality control the broth and the solid media, and to quality control antibiotics during susceptibility testing.

**Data management and statistical analysis:** demographic data for each participant was entered on a paper-based data collection tool. Microbial data was extracted from the laboratory registry into a data collecting tool. An electronic data entry spreadsheet was created in Excel. The data was then entered from the data collecting tool into an Excel spreadsheet. The data set was cleaned for missing variables and duplicate entries and out-of-range values. Data analysis was done using Epi info (version 7.2.1.0). Demographic data and bacteria isolated and identified with their corresponding antimicrobial resistance patterns were analyzed using proportions and percentages. Distributions of prevalence were compared across wards and professionals using cross-tabulations.

**Ethics approval:** the IRB of the School of Biomedical Sciences Makerere University College of Health Sciences approved the study protocol (Approval Number: SBS-361).

## Results

**Figure 1:**
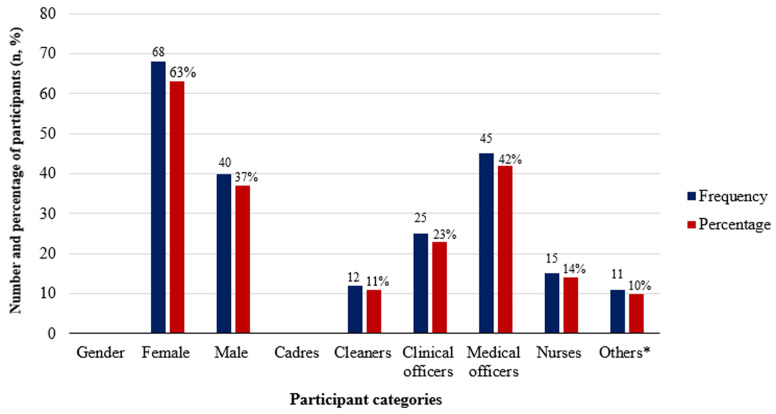
demographic characteristics of participant health care workers; *others include administrators, pharmacists, and security personnel

**Demographic data:** one hundred and eight (108) HCWs were included in this study. The demographic characteristics of participant HCWs are shown in Figure 1. Sixty-eight (63%) were female while 40 (37%) were male. Study participants' age ranged from 21 to 59 years, with a mean age of (33.79 SD ± 8.78). Of the 108 study participants, 12 (11.1%) were cleaners, 11 (10.2%) were clinical officers, 25 (23.1%) were medical officers, 45 (41.7%) nurses and 15 (13.9%) were others (administrators, pharmacists, and security personnel).

**Figure 2:**
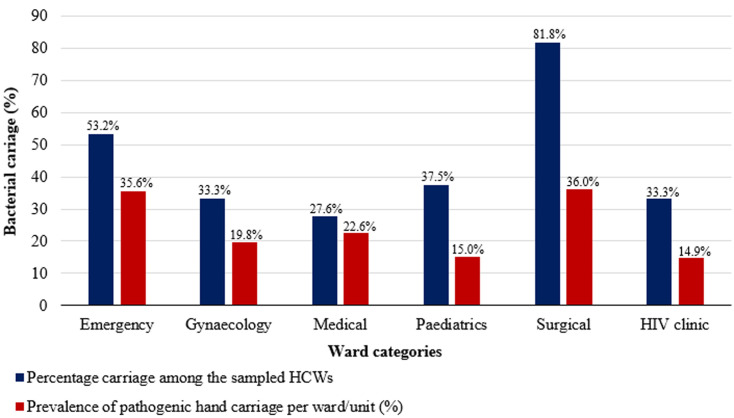
prevalence of bacterial carriage among healthcare workers by ward

**Prevalence of bacterial carriage among health care workers by ward:** the overall carriage of at least one species of pathogenic bacteria among participants was 42.6% (46/108). Figure 2 and Table 1 show the prevalence of bacterial carriage among HCWs by ward. Percentage carriage among sampled HCWs per ward/unit was surgical ward 81.8% (9/11) emergency unit 53.2% (16/30), pediatrics ward 37.5% (3/8), gynecology ward 33.3% (7/21), HIV clinic 33.3% (3/9) and medical ward 27.6% (8/29). The prevalence of hand carriage was significantly associated with the ward/unit, a p-value of 0.009. The rest of the prevalence carriage is shown in Table 2.

**Table 1:** bacterial carriage among HCWs on different wards

Ward	HCWs sampled (N)	HCWs with no growth n (%)	HCWs with growth n (%)
Emergency	30	14 (22.6)	16 (34.8)
Genecology	21	14 (22.6)	7 (15.2)
Medical	29	21 (33.8)	8 (17.4)
Paediatrics	8	5 (8.1)	3 (6.5)
Surgical	11	2 (3.2)	9 (19.6)
HIV clinic	9	6 (9.7)	3 (6.5)
Total	108	62 (57.4)	46 (42.6)

HCWs: health care workers

**Table 2:** bacterial carriage of different wards or units: the proportions, prevalence, and comparison of rate of carriage for all the wards

Wards/units	Participants (N=108)	Positive (N=46)	Percentage carriage among the sampled HCWs (%)	Number of HCWs in each ward/unit	Proportion of HCWs in the ward that were sampled	Prevalence of pathogenic hand carriage per ward/unit	Rate of pathogenic hand carriage compared to reference (lowest prevalence)
Emergency	30	16	53.2	45	0.67	35.6	2.3
Gynaecology	21	7	33.3	35	0.60	19.8	1.3
Medical	29	8	27.6	35	0.82	22.6	1.5
Paediatrics	8	3	37.5	20	0.40	15.0	1.0
Surgical	11	9	81.8	25	0.44	36.0	2.4
HIV clinic	9	3	33.3	20	0.45	14.9	0.5

**Figure 3:**
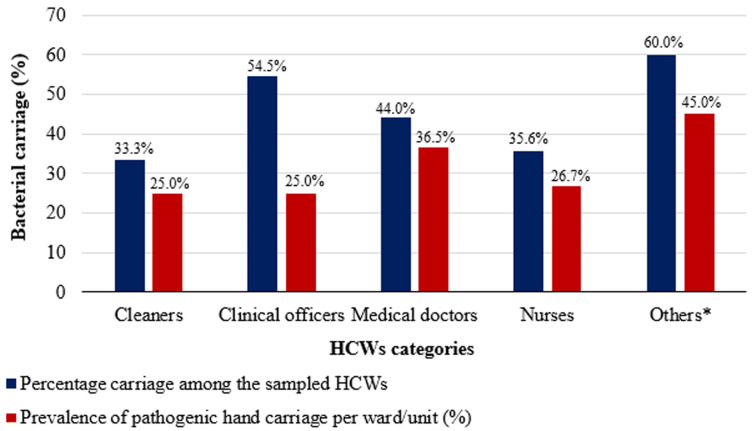
prevalence of bacterial carriage by professional category; *others include administrators, pharmacists, and security personnel

**Prevalence of bacterial carriage by professional category:** the percentage carriage per category of HCWs were others 9 (60%), clinical officers 6 (54.5%), medical doctors 11 (44%), nurses 16 (35.6%), cleaners 4 (33.3%). However, bacterial contamination of HCWs´ hands was not associated with any profession, p-value of 0.838. Figure 3 and Table 3 show the summarised proportions and carriage rates among different professional categories.

**Table 3:** bacterial carriage of different professional categories: the proportions, prevalence, and comparison of rate of carriage for the professional categories

Wards/units	Participants (N=108)	Positive (N=46)	Percentage carriage among the sampled HCWs (%)	Number of HCWs in each ward/unit	Proportion of HCWs in the ward that were sampled	Prevalence of pathogenic hand carriage per ward/unit	Rate of pathogenic hand carriage compared to reference (lowest prevalence)
Cleaners	12	4	33.3	16	0.75	25	Ref
Clinical officers	11	6	54.5	24	0.46	25	1
Medical doctors	25	11	44.0	30	0.83	36.5	1.5
Nurses	45	16	35.6	60	0.75	26.7	1.1
Others	15	9	60.0	20	0.75	45	1.8

**Figure 4:**
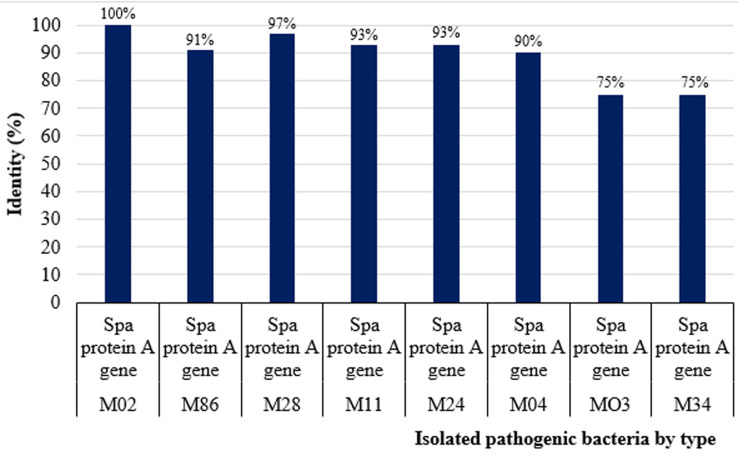
percentage identity of isolated pathogenic bacteria by type

**Types of pathogenic bacteria isolated:** the total pathogenic bacteria obtained from the 46 HCWs were 69 isolates. Figure 4 shows the percentage identity of isolated pathogenic bacteria by type. Out of the 69 isolates, 20/69 (29%) were *S. aureus* and 15/69 (21.7%) were *E. coli*. Other significant isolates included: *Acinetobacter spp*. 10/69 (14.5%), *Klebsiella spp*. 10/69 (14.5%), *Enterococcus spp* 4/69 (5.8%), *Citrobacter spp* 2/69 (3%), *Providencia spp* 2/69 (3%), *Enterobacter spp*. 1/69 (1.4%), *Morganella morgnii*, 1/69 (1.4%), *Streptococcus viridians* 1/69 (1.4%) and *Pseudomonas spp*. 3/69 (4.3%).

**Figure 5:**
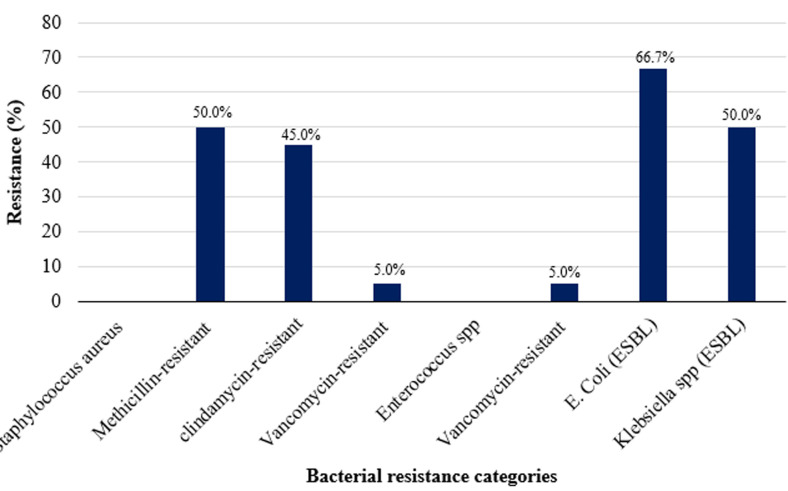
prevalence of resistant isolates percentage

**Resistance patterns of isolated bacteria:** Figure 5 shows the resistance patterns of isolated bacteria. Of the *Staphylococcus aureus* isolated, 50% were methicillin-resistant (MRSA), 45% had inducible clindamycin resistance (D test positive) and 5% were resistant to vancomycin (VRSA). Among the *Enterococcus spp*. 50% of isolates were resistant to vancomycin. For the Gram-negative bacteria, 10 (66.7%) of *E. coli* were phenotypically found to be ESBL-producing. In addition to producing ESBL, 2 isolates of *E. coli* were found to produce AmpC beta-lactamases. Screening of the *E. coli* isolates for carbapenemase showed no organisms with carbapenemase activity and no Metallo-beta lactamases (MBL) activity. Fifty (50%) of *Klebsiella spp*. were found to produce ESBL. However, on screening, they did not carry AmpC and MBL enzymatic activities.

**Relatedness among predominant ESBL (*E. coli*):** Following ERIC PCR and bionumeric analysis, 3 clades of *E. coli* were revealed. Clade-1 with isolates 34 and 80. Clade-2 isolates 32, 44, 47 and clade-3 with isolates 21 and 12. All 10 isolates had similar antibiotic susceptibility patterns for non-beta lactam antibiotics. They were all resistant to ciprofloxacin, tetracycline and cotrimoxazole. Susceptibility variation to chloramphenicol was noted at 50% (5/10). Similar patterns were noted within clade-1 and clade-3.

**Spa typing of methicillin-resistant *Staphylococcus aureus*:** of all MRSA screened, 8/10 (80%) isolates had analyzable sequences whereas 2/10 (20%) were bad sequences as shown in Table 4. Attempts to determine spatypes identified one isolate as t335 and the rest were not defined by the spaTyper database.

**Table 4:** BLAST and spa-type results of MRSA isolates from different wards

Isolate ID	BLAST (blastn) results	E-value	Percentage identity (%)	Spa type	Ward
**M02**	Spa protein A gene	8e-154	100	t335	Surgical
**M86**	Spa protein A gene	2e-82	91	Not defined^*^	Gynaecology
**M28**	Spa protein A gene	2e-101	97	Not defined^*^	Emergency
**M11**	Spa protein A gene	9e-138	93	Not defined^*^	Surgical
**M24**	Spa protein A gene	2e-69	93	Not defined^*^	Medical
**M04**	Spa protein A gene	8e-40	90	Not defined^*^	Surgical
**MO3**	Spa protein A gene	9e-85	75	Not defined ^µ^	Surgical
**M34**	Spa protein A gene	4e-134	75	Not defined ^µ^	Emergency
**M57**	Bad sequence	N/A	N/A	Not defined	Emergency
**M10^¶^**	Bad sequence	N/A	N/A	Not defined	Surgical

* These were likely to be a surface protein A gene but not defined by the Spatyper database ^µ^These were likely to be a Surface protein A genes but not defined by the Spatyper database

¶ These isolates had bad sequences that were not analysable

**Figure 6:**
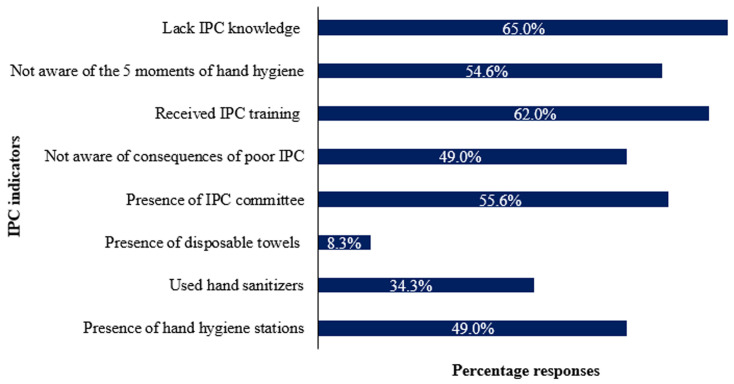
percentage response among surveyed healthcare workers

**Infection prevention and control program, hand hygiene knowledge and infrastructure:** Figure 6 shows the responses of surveyed HCWs. Among the 108 participants in this study, only 49% reported the presence of hand hygiene stations at the point of care. Thirty-seven participants 37 (34.3%) reported having used hand sanitizers, the majority of whom (80%) were medical doctors from the HIV clinic and medical wards. Also, only 9 (8.3%) of the participants reported the availability of disposable towels or paper tissue at hand washing stations. Fifty-five percent (55.6%) of participants recognized the presence of an infection control committee in the hospital. Forty-nine percent 49% of all participants were not aware of problems associated with poor infection control measures. Sixty-seven, 67 (62%) of the participants reported receiving IPC training, with the majority (75%) of staff that had received IPC training from the HIV clinic. Fifty-four percent (54.6%) of the HCWs were not aware of the moments of hand hygiene. Sixty-five (65%) of participants lacked infection control knowledge.

## Discussion

This cross-sectional study found a high carriage of pathogenic bacteria among HCWs in the study site. Furthermore, there was a variation in the carriage of bacteria across the hospital wards and among health worker professional categories. The bacterial carriage was highest among clinical officers, followed by doctors, cleaners, and nurses respectively. Among wards, HCWs in the surgical wards had the highest bacterial carriage, followed by the emergency department and pediatrics ward, respectively. The isolated bacteria were predominantly gram-negative, with *E. coli* the most frequently isolated bacteria followed by *Acinetobacter spp* and *Klebsiella spp. S. aureus* was the most frequently isolated gram-positive bacteria. The majority of the bacteria were MDR with MRSA, VRSA and ESBL identified among the isolates. Programmatic assessment of IPC practices found lack of a hospital IPC program, low knowledge of hand hygiene and lack of training in hand hygiene practice at the hospital. The role of HCWs' hands in the sustained transmission of nosocomial infections has been previously reported [17,18]. In this study, 42.6% of HCWs were found to carry pathogenic bacteria on their hands. Our study findings were similar to those from other tertiary care hospitals, in resource-limited settings that found a carriage similar levels of bacterial carriage [19-21]. The findings from these low-income settings resonate with those in our study given similarities in health care systems and challenges. However, some studies from high-income countries have shown a lower bacterial carriage by HCWs [17,22].

The pathogenic bacteria isolated in this study were also similar to findings in other studies that have shown that *Staphylococcus aureus, Enterobacter species, Acinetobacter, Klebsiella spp., E. coli, Morganella morgana*, and *Pseudomonas aeruginosa* as the most common bacteria carried by HCWs [17,19,23]. In our study, significantly high antibiotics resistance patterns were seen among all isolates to commonly used antibiotics (ampicillin, trimethoprim-sulphamethoxazole and tetracycline) in both gram negatives and gram positives, with MRSA, VRSA and ESBL reported, a finding similar to other studies [5,24-26]. The heterogenic nature of pathogenic bacteria identified in our study (causation, variation among HCWs, carriage and resistance) was similar to findings in a recent systematic review [27].

Genotyping ESBL *E. coli* by ERIC PCR revealed cross-transmission of related bacteria around different wards. This could be due to the transfer of these pathogenic bacteria from the emergency ward to both surgical and medical wards. This study finding is probably the first to reveal a transmission chain within a hospital setting using pathogens from HCWs' hands in Uganda. Of the 10 MRSA isolates, one of the spa type (t355) that was circulating in the surgical ward has previously been described in the surgical ward at Mulago Hospital [5]. This raises the possibility of intra-health facility transmission of pathogenic bacteria in Uganda, a finding that has been described in other settings and as a risk factor for nosocomial infections [28-31]. The finding of bacterial carriage among all HCWs, including cleaners and variation among wards is similar to the finding of heterogeneity of bacterial carriage among HCWs in a recent systematic review [27]. The higher carriage rates of pathogenic bacteria in the emergency unit were probably due to the high workload as HCWs attend to emergency cases hence compromising adherence to hand washing.

Knowledge of the role of IPC programs was higher among nurses than doctors. This is similar to findings from another study done by Sethi *et al*. at Mulago national referral hospital [32]. This could be due to the high level of training in IPC among nurses compared to doctors. Lack of IPC supplies, shortage of HCWs in the emergency ward, lack of soap and water, and alcohol hand rub stockouts were the main factors affecting hand hygiene. These factors were similar to other contributing factors to low IPC practice in health facilities in settings similar to Uganda [33-38].

**Strengths and limitations:** a key strength of our study is that all HCWs at the hospital participated in the study. A limitation is that the number of bacteria identified is not high enough for each of the bacteria to inform susceptibility patterns since a minimum of 30 isolates from each bacteria is needed for resistance patterns for treatment purposes. Lastly, molecular typing of isolates from patients and HCWs for comparison was not done to determine if admitted patients were suffering from HAIs like bacteria carried by the HCWs.

## Conclusion

The findings of this study indicate that endemic pathogenic bacterial hand carriage among HCWs at Naguru Regional Referral Hospital is highly significant, with most of the bacterial multiple drug resistant (MDR). The finding of low hand practice of IPC coupled with high pathogenic bacterial carriage among HCWs is a public health concern. These findings warrant the urgent need to invest in hospital IPC programs, with a focus on hand hygiene to combat AMR and improve patient safety.

### What is known about this topic

- *Drug-resistant infections are a leading cause of mortality, with 4.95 million attributable deaths in 2019 alone;*
- *Health care workers can be colonized and act as a vehicle for the transmission of drug-resistant infections in hospital settings;*
- *The uptake of infection prevention and control practices remains low in many resources limited settings, which predisposes patients to a risk of healthcare-associated infections*.

### What this study adds

- *This study adds to evidence that bacteria carriage by health care workers in the study health facility were related between health workers and were drug-resistant;*
- *The study shows a high rate of bacterial carriage by health workers and low levels of training in IPC and awareness of IPC in the study hospital*.
